# Trends in long term vaping among adults in England, 2013-23: population based study

**DOI:** 10.1136/bmj-2023-079016

**Published:** 2024-07-17

**Authors:** Sarah E Jackson, Harry Tattan-Birch, Lion Shahab, Jamie Brown

**Affiliations:** 1 Department of Behavioural Science and Health, University College London, London, UK; 2 SPECTRUM Consortium, Edinburgh, UK

## Abstract

**Objective:**

To examine trends in long term (>6 months) vaping among adults in England.

**Design:**

Population based study.

**Setting:**

England.

**Participants:**

179 725 adults (≥18 years) surveyed between October 2013 and October 2023.

**Main outcome measures:**

Time trends in prevalence of long term vaping using logistic regression, overall and by vaping frequency (daily or non-daily), and main type of device used (disposable, refillable, or pod).

**Results:**

The proportion of adults reporting long term vaping increased non-linearly, from 1.3% (95% confidence interval 1.1% to 1.5%) in October 2013 to 10.0% (9.2% to 10.9%) in October 2023, with a particularly pronounced rise from 2021. This rise included an increase in long term daily vaping, from 0.6% (0.5% to 0.8%) to 6.7% (6.0% to 7.4%). Absolute increases in long term vaping were larger among people with a history of regular smoking (current smokers: 4.8% (4.0% to 5.8%) to 23.1% (20.4% to 25.9%); recent former smokers: 5.7% (3.4% to 9.2%) to 36.1% (27.6% to 45.4%); long term former smokers: 1.4% (1.0% to 1.9%) to 16.2% (14.2% to 18.4%)), but an increase also occurred among people who had never regularly smoked (0.1% (0.0% to 0.2%) to 3.0% (2.3% to 3.8%)). Growth was also more pronounced in young adults (eg, reaching 22.7% (19.2% to 26.5%) of 18 year olds *v* 4.3% (3.6% to 5.2%) of 65 year olds), including among those who had never regularly smoked (reaching 16.1% (11.1% to 22.7%) of 18 year olds *v* 0.3% (0.1% to 0.6%) of 65 year olds). Between October 2013 and March 2021, most long term vapers mainly or exclusively used refillable electronic cigarettes (2.5% to 3.3% of adults) and few (0.1% of adults) used disposable devices. However, prevalence of long term vaping using disposable devices subsequently rose rapidly, and by October 2023 similar proportions of adults mainly or exclusively used disposable and refillable devices (4.9% (4.2% to 5.7%) and 4.6% (4.0% to 5.3%), respectively).

**Conclusions:**

The prevalence of long term vaping increased substantially among adults in England during 2013-23. Much of this increase occurred from 2021, coinciding with the rise in popularity of disposable e-cigarettes. Half of long term vapers now mainly or exclusively use disposable devices. The growth was concentrated among people with a history of regular smoking, but an increase also occurred among people who never regularly smoked, especially young adults.

## Introduction

Electronic cigarettes are an effective tool for smoking cessation^1^ ^2^ and are likely much less harmful than conventional cigarettes.^3^ They are not, however, risk-free, particularly for people who have never smoked.^3^ As such, a challenge many countries currently face is how to regulate e-cigarettes so that they are available and appealing to smokers as a means to quit smoking while minimising uptake among those who would not have otherwise smoked. Until recently, England appeared to have struck the right balance, with e-cigarettes widely used by smokers in quit attempts^4^ and rarely used by young people and never smokers.^5^ ^6^ However, the prevalence of vaping has risen in recent years, particularly among adolescents (11-17 years)^5^ and young adults (18-24 years).^4^ ^6^ This increase in prevalence has largely been attributed to the introduction of new disposable devices.^7^ ^8^ It is not clear how far this rise reflects an increase in short term, experimental use versus sustained, regular use. In addition, little is known about how the types of products used by long term vapers—and how often they use them—is changing over time. Understanding this is important for gauging the likely public health and environmental effects of this rise in vaping uptake and for informing policy decisions.

The use of e-cigarettes increased rapidly among adolescents and young adults in the US between 2015 and 2019, resulting in widespread concern about public health.^9^ ^10^ Further analyses of the data showed that much of the use was experimental,^11^ and evidence of an overall increase in the population burden of nicotine dependence was limited.^12^ Consistent with this pattern of use, and following new regulations restricting the availability of e-cigarettes, the prevalence of use in the same population subsequently declined.^13^ Recently in the UK, vaping has increased noticeably among adolescents and young adults.^5^ ^7^ It is important to understand the extent to which this represents experimental or dependent use, and how this differs across subgroups of the population (eg, by smoking status, age, gender, and socioeconomic position). One indicator of more dependent use is the prevalence of long term vaping.

Long term vaping could be harm reducing or harmful to public health, depending on who is using the products and what they would otherwise be doing. Transitioning to long term vaping from cigarette smoking—the most harmful form of nicotine use—would reduce harm for people who are unable or unwilling to stop all nicotine use.^3^ Conversely, long term vaping could lead to harm among those who would have never started smoking cigarettes.^3^ Long term vaping with disposable e-cigarettes specifically may also have a substantial environmental impact^14^: these products are designed for single use, so they generate more waste than rechargeable vaping products (ie, refillable and pod devices).^15^ ^16^

The Smoking Toolkit Study (a nationally representative, cross sectional survey) collects detailed data on vaping among adults in England each month. In the current study we used data collected from 2013 to 2023 to examine how the prevalence of long term (>6 months) vaping changed overall and by vaping frequency (daily or non-daily) in adults during this period, and the main type of device used (disposable, refillable, or pod). We also investigated whether changes in any long term vaping and in long term vaping using a disposable device differed by smoking status, age, gender, and occupational social grade.

## Methods

### Pre-registration

The study protocol and analysis plan were pre-registered on Open Science Framework (https://osf.io/n2785/). We also carried out additional unplanned analyses before and after peer review (see statistical analysis section).

### Design

Data were drawn from the ongoing Smoking Toolkit Study, a monthly cross sectional survey of a representative sample of adults in England.^17^ The study uses a hybrid of random probability and simple quota sampling to select a new sample of about 1700 teenagers and adults (≥16 years) each month. Comparisons with sales data and other national surveys indicate that key variables, including sociodemographic characteristics, smoking prevalence, and cigarette consumption, are nationally representative.^17^ ^18^

The methods have been described in detail elsewhere.^17^ ^19^ Briefly, England is split into 165 665 output areas, each comprising about 300 households. These areas are then stratified according to established geodemographic characteristics and geographical region, then randomly selected into an interviewer’s list.

Before the covid-19 pandemic these areas were randomly allocated to interviewers who travelled to their selected areas to engage with one household member aged ≥16 years. Face-to-face computer assisted interviews were conducted until quotas based on factors influencing the probability of being at home (ie, working status, age, and gender) were fulfilled. Morning interviews were avoided to maximise the availability of participants.

In-person interviews were halted in March 2020 owing to covid-19 social distancing restrictions and replaced by telephone interviews from April 2020 onwards with adults aged ≥18 years. The lower age limit returned to 16 years from January 2022. Telephone interviews are now conducted by landline or mobile using random digit dialling or by targeted mobile. For the sample processed, each eligible landline telephone number across England has a random probability of selection proportionate to population distribution (ie, stratification of the landline telephone database by and within Government Office Region; see^19^ for further details). To maximise response rates, sampling takes place mostly by landline earlier in the day and mostly by mobile phone later in the day.

The two methods of data collection show good comparability: when social distancing restrictions were lifted, we ran a parallel telephone and face-to-face survey wave and obtained similar estimates for key sociodemographic, smoking, and nicotine product use measures.^20^

For this study, we used data collected from participants surveyed between October 2013 (the first wave to assess vaping among all adults) and October 2023 (the most recent data at the time of analysis). Since April 2022, vaping duration and device characteristics have only been assessed quarterly as a means of reducing the cost of data collection for the Smoking Toolkit Study owing to limited funding. We excluded waves in which vaping duration were not assessed (May, June, August, September, November, and December 2022, and February, March, May, August, and September 2023); trends were modelled based on data collected in all other waves. Data on the main device type used have only been collected since July 2016, so trends in this outcome were limited to the period from July 2016 to October 2023. Because data were not collected from teenagers aged 16 and 17 years between April 2020 and December 2021, we restricted our sample to those aged ≥18 years for consistency across the time series.

### Measures

#### Long term vaping

Long term vaping was defined as current vaping for a period of more than six months. Current vaping was assessed within several questions asking about use of a range of nicotine products, depending on the participant’s smoking status. Current smokers were asked: “Which, if any, of the following are you currently using to help you cut down the amount you smoke?” and “Do you regularly use any of the following in situations when you are not allowed to smoke?”; current smokers and those who had quit in the past year were asked: “Can I check, are you using any of the following either to help you stop smoking, to help you cut down or for any other reason at all?”; and non-smokers were asked: “Can I check, are you using any of the following?” We considered those who reported using an e-cigarette (ie, selected the response option “electronic cigarette” or “Juul”) in response to any of these questions to be current vapers. Other response options included different types of nicotine replacement therapy (eg, nicotine gum, nicotine patch), heated tobacco products (heat-not-burn cigarette (eg, iQOS, heatsticks)), and nicotine pouches (tobacco-free nicotine pouch or pod or “white pouches” placed on the gum). The supplementary file provides details of these items (including an additional question on nicotine vaping assessed in a subset of waves). These items capture all vaping rather than just nicotine vaping, although most vapers (87%) sampled in waves that assessed nicotine content said that their usual device contained nicotine.

Current vapers were asked: “How long have you been using this nicotine replacement product or these products for?” Response options were <1 week, 1-6 weeks, >6-12 weeks, >12-26 weeks, >26-52 weeks, and >52 weeks.

Participants who reported vaping for more than six months were considered long term vapers.^21^ This definition was used to indicate possible dependent use. Because this measure for duration of use was not specific to vaping but applied to all non-combustible nicotine products the participant reported using, we conducted a sensitivity analysis in which we restricted the definition of long term vaping to those reporting no current use of nicotine replacement therapy, heated tobacco products, or nicotine pouches.

#### Vaping frequency

Vaping frequency was assessed by asking vapers: “How many times per day on average do you use your nicotine replacement product or products?” Response options were 1, 2, 3-4, 5-7, 8-11, ≥12, not every day but at least once a week, not every day and less than once a week, and don’t know.

We considered those who reported use at least once a day to be vaping daily and those who reported use less than once a day to be vaping non-daily. Because this measure was not specific to vaping, we conducted a sensitivity analysis restricting the definition of daily and non-daily vaping to those reporting no current use of nicotine replacement therapy, heated tobacco products, or nicotine pouches.

#### Main device type

We assessed the main type of device used from July 2016 onwards by asking vapers: “Which of the following do you mainly use . . .?” Response options were disposable (“A disposable e-cigarette or vaping device (non-rechargeable)”), refillable (“An e-cigarette or vaping device with a tank that you refill with liquids (rechargeable)” or “A modular system that you refill with liquids (you use your own combination of separate devices: batteries, atomizers, etc.)”), and pod (“An e-cigarette or vaping device that uses replaceable pre-filled cartridges (rechargeable).”)

#### Smoking status

Smoking status was assessed by asking participants which of the following best applied to them: I smoke cigarettes (including hand-rolled) every day; I smoke cigarettes (including hand-rolled), but not every day; I do not smoke cigarettes at all, but I do smoke tobacco of some kind (eg, pipe, cigar, or shisha); I have stopped smoking completely in the last year; I stopped smoking completely more than a year ago; and I have never been a smoker (ie, smoked for a year or more).

Those who responded to the first three of these options were considered current smokers; those who responded they had stopped smoking in the past year were considered recent former smokers; and those who responded they had stopped smoking more than a year ago were considered long term former smokers. Those who responded they had never been a smoker were considered never-smokers, and given the wording of the response options, this group would capture some people who have smoked at all in the past. However, our question does ask people to choose which of the response options best applies to them; the “smoked for a year or more” is an elaboration rather than a definition. As such, it is likely that people who have smoked cigarettes with any kind of recent frequency (regardless of whether it is more than a year) are more likely to respond to having smoked cigarettes (including hand-rolled) every day or not every day (if currently doing so) or to have stopped smoking completely in the past year (if they have recently stopped). Other national surveys use alternative definitions, but the surveys have produced consistently similar estimates of prevalence, and the Smoking Toolkit Study and sales data are also closely aligned.^22^

#### Sociodemographic characteristics

We modelled age as a continuous variable using restricted cubic splines. Descriptive data were also provided by age group (18-24, 25-34, 35-44, 45-54, 55-64, and ≥65 years).

Gender was self-reported as man or woman. In more recent waves, participants have also had the option to describe their gender in another way; those who identified in another way were excluded from analyses by gender owing to low numbers.

Occupational social grade was categorised as ABC1 (includes managerial, professional, and upper supervisory occupations) and C2DE (includes manual routine, semi-routine, lower supervisory, state pension, and long term unemployed). This occupational measure of social grade is a valid index of socioeconomic position that is widely used in research in UK populations. It has been identified as particularly relevant in the context of tobacco use.^23^

### Statistical analysis

Data were analysed in R version 4.2.1. The Smoking Toolkit Study uses raking to weight the sample to match the population in England. This profile is determined each month by combining data from the UK Census, the Office for National Statistics mid-year estimates, and the National Readership Survey.^17^ The following analyses used weighted data. Missing cases were excluded on a per analysis basis.

#### Trends in long term vaping among adults

Among all adults, we reported the prevalence and corresponding 95% confidence interval (CI) of six long term vaping categories by survey year: vaping (>6 months), daily vaping (>6 months, and currently vaping daily), non-daily vaping (>6 months, and currently vaping non-daily), vaping using a disposable device (>6 months, and currently mainly or exclusively using disposable e-cigarettes), vaping using a refillable device (>6 months, and currently mainly or exclusively using refillable e-cigarettes), and vaping using a pod device (>6 months, and currently mainly or exclusively using pod e-cigarettes).

We used logistic regression to analyse trends in these long term vaping outcomes over the study period, with time (survey month) modelled using restricted cubic splines with five knots (decided a priori and pre-registered, on the basis that this number would be sufficient to accurately model trends across years without overfitting; in cubic spline models, the results are robust to the specific position and number of knots (at least when choosing between four or five knots)^24^). This approach allowed for flexible and non-linear changes over time, while avoiding categorisation.

#### Differences by smoking status, age, gender, and occupational social grade

To explore moderation of trends in any long term vaping and in long term vaping using a disposable device by smoking status, age, gender, and occupational social grade, we repeated these models including the interaction between the moderator of interest and time—thus allowing for time trends to differ across subgroups. Each of the interactions was tested in a separate model. Age was modelled using restricted cubic splines with three knots (placed at the 5%, 50%, and 95% quantiles), to allow for a non-linear association between age and long term vaping.

We used predicted estimates from these models to plot the prevalence of long term vaping over the study period among all adults and within each subgroup of interest. As age was modelled continuously using splines, we displayed estimates for six specific years of age (those aged exactly 18, 25, 35, 45, 55, and 65 years) to illustrate how trends differed across the age spectrum. The model used to derive these estimates included data from participants of all ages, not only those with the previous exact specified ages.

#### Unplanned analyses

Before peer review, we modelled age specific trends in long term vaping (ie, repeated the model testing the interaction between age and time) among participants who had never regularly smoked—those who responded that they had never been a smoker (ie, smoked for a year or more). This allowed us to examine the extent to which the increase in long term vaping we observed among never smokers differed by age.

After peer review, we added five further unplanned analyses. The first was a segmented regression analysis that tested the association of the rise in popularity of vaping using disposable devices among young adults in England with changes in the trend in long term vaping. Previous data from the Smoking Toolkit Study indicated that disposable e-cigarette use in Great Britain was relatively rare (<1%) up to May 2021, but rose gradually to 2% (11% among 18 year olds) by April 2022.^7^ We therefore split the time series into two periods: October 2013 to May 2021 (pre-disposables period) and June 2021 to October 2023 (disposables period). We used logistic regression to model the trend in long term vaping before the interruption (underlying secular trend; coded 1 . . . *n*, where *n* was the total number of waves) and the change in the trend (slope) post-disposables relative to pre-disposables (coded 0 before the rise in disposable e-cigarette use, and 1 . . . *m* from June 2021 onwards, where *m* was the number of waves after June 2021). We assumed linear trends pre-interruption and post-interruption. The model was adjusted for seasonality, which was modelled using a smoothing term with cyclic cubic splines specified. We also adjusted for the onset of the covid-19 pandemic (coded 0 to February 2020 and 1 from March 2020), to account for any influence of the pandemic on vaping patterns. For ease of interpretation, we multiplied coefficients by 12 to convert results from monthly to annual trends. 

The second unplanned analysis added after the peer review repeated our (spline and segmented regression) trend analyses restricting the definition of long term vaping to those who did not also use other non-combustible nicotine products, to explore the possible influence of the measures assessing duration and frequency of use not referring exclusively to vaping. The third repeated our primary (spline) models for long term daily vaping and long term non-daily vaping among participants who had never regularly smoked, to explore whether the growth in vaping among this subgroup was predominantly daily or non-daily. The fourth repeated our primary models for long term vaping, among all adults and by age, with adjustment for psychological distress, to explore the extent to which the rise in long term vaping we observed may have been driven by rising levels of distress in the population over this period, particularly among young adults.^25^ Because data on psychological distress were only collected between April 2020 and June 2023, these analyses were restricted to participants surveyed during this period, and we reduced the number of knots used to model time from five to three to avoid overfitting the data (given the much shorter time period). The fifth provided detailed descriptive data on vaping frequency among long term vapers (ie, the proportion selecting each response option for the item assessing vaping frequency), to provide more information beyond daily versus non-daily use.

### Patient and public involvement

The wider Smoking Toolkit Study is discussed several times a year with a diverse patient and public involvement group, and the authors regularly attend and present at meetings at which patients and members of the public are included. Interaction and discussion at these events help shape the broad research priorities and questions. A mechanism also exists for generalised input from the wider public: each month, interviewers seek feedback on the questions from all respondents, who are representative of the English population. This feedback is limited and usually relates to understanding of questions and item options. No patients or members of the public were involved in setting the research questions or the outcome measures, nor were they involved in the design and implementation of this specific study.

## Results

A total of 197 266 (unweighted) adults aged ≥18 years in England were surveyed between October 2013 and October 2023. We excluded 17 541 surveyed in months in which vaping duration was not assessed, resulting in a final analytical sample of 179 725 participants. Of these, 125 751 were surveyed between July 2016 and October 2023 and provided data for analyses by the main type of vaping device used. Supplementary table S1 shows the characteristics of the whole analysed sample and the subsample analysed by device type.

### Trends in long term vaping among adults

**Fig 1:**
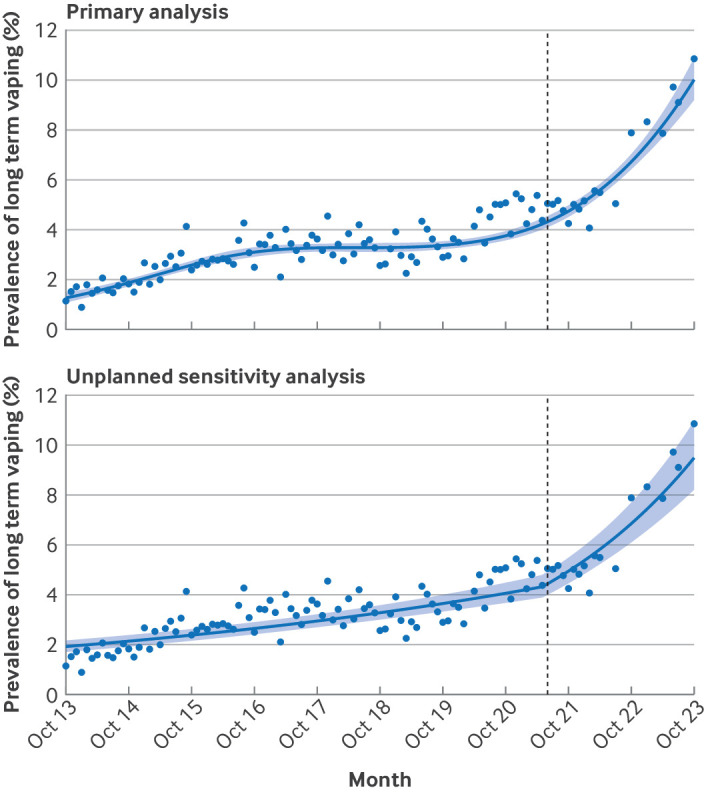
Trends in long term vaping among adults in England, October 2013 to October 2023. In the primary model, the survey wave was modelled non-linearly using restricted cubic splines (five knots). The unplanned sensitivity analysis used a segmented regression approach to model associations of the start of the rise in popularity of disposable electronic cigarettes with a change in the trend of long term vaping (adjusted for seasonality and the onset of the covid-19 pandemic). Lines represent modelled weighted prevalence by monthly survey wave. Shaded bands represent 95% confidence intervals. Points represent unmodelled weighted prevalence by month. Vertical dashed line indicates start of the rise in popularity of vaping using disposable devices in June 2021

Our primary model indicated that across the study period, the proportion of adults reporting long term vaping increased from 1.3% to 10.0% (table 1). The increase over time was non-linear: prevalence increased from 1.3% to 3.3% between October 2013 and July 2017, was stable at 3.3% between July 2017 and August 2019, then increased again—with a particularly sharp rise from late 2021—reaching 10.0% by October 2023 (fig 1).

**Table 1:** Modelled estimates of prevalence of long term vaping among adults in England at start and end of study period, overall and by vaping frequency and main type of device used

Outcomes for long term vaping	Prevalence, % (95% CI)*		
	October 2013	July 2016	October 2023
Overall	1.3 (1.1 to 1.5)	3.0 (2.8 to 3.2)	10.0 (9.2 to 10.9)
By vaping frequency†:			
Daily vaping	0.6 (0.5 to 0.8)	2.1 (1.9 to 2.3)	6.7 (6.0 to 7.4)
Non-daily vaping	0.6 (0.5 to 0.8)	0.7 (0.6 to 0.8)	1.6 (1.3 to 2.0)
By main device type used†:			
Disposable	–	0.1 (0.0 to 0.2)	4.9 (4.2 to 5.7)
Refillable	–	2.5 (2.1 to 2.8)	4.6 (4.0 to 5.3)
Pod	–	0.3 (0.2 to 0.5)	1.0 (0.8 to 1.3)

CI=confidence interval.

* Data for October 2013, July 2016, and October 2023 are weighted estimates of prevalence in these months from logistic regression, with survey month modelled non-linearly using restricted cubic splines (five knots). October 2013 and October 2023 were the first and last months in the time series, and July 2016 was the first month in which the main device type used by vapers was assessed.

† Prevalence estimates do not sum to total prevalence of long term vaping owing to missing data (participants did not respond or responded don’t know) and each trend being modelled separately. Modelled estimates by device type sum to slightly more than the total modelled estimate, but in the unmodelled data (supplementary table S2) estimates by device type consistently sum to slightly less than the total estimate (consistent with a small deficit owing to missing data).

Supplementary table S2 provides unmodelled estimates of prevalence within each survey year.

The unplanned segmented regression analysis was consistent with this rise being associated with an increase in popularity of disposable e-cigarettes (fig 1). Before June 2021, the prevalence of long term vaping increased by 11.3% per year (relative risk for trend 1.113 (95% CI 1.092 to 1.133)). However, this trend increased sharply from when disposable e-cigarettes became popular (relative risk for trend change 1.245 (1.181 to 1.312)), with prevalence rising by 38.6% per year since (relative risk for trend×relative risk for trend change 1.113×1.245=1.386). These percentages represent the yearly relative rather than absolute percentage point increase.

**Fig 2:**
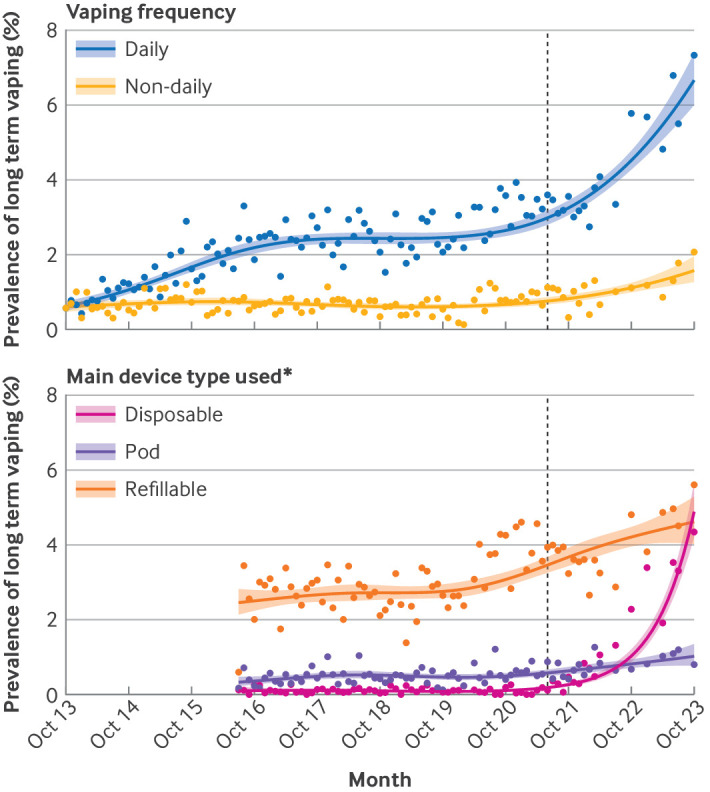
Trends in long term vaping by frequency and main device type among adults in England, October 2013 to October 2023. Lines represent modelled weighted prevalence by monthly survey wave, modelled non-linearly using restricted cubic splines (five knots). Shaded bands represent 95% confidence intervals. Points represent unmodelled weighted prevalence by month. Vertical dashed line indicates start of the rise in popularity of vaping using disposable devices in June 2021. *Not assessed before July 2016. Prevalence estimates by vaping frequency and main device type used do not sum to the total prevalence of long term vaping owing to some missing data (participants either did not respond or responded that they did not know) and each trend being modelled separately

A greater increase occurred in long term daily vaping than long term non-daily vaping over time (table 1 and supplementary table S2). In October 2013, equal proportions of long term vapers reported vaping daily and non-daily (0.6% and 0.6% of adults, respectively; table 1; estimates do not sum to the total prevalence of long term vaping owing to some missing data on vaping frequency). The trend in long term daily vaping mirrored the trend in any long term daily vaping, increasing to 6.8% of adults by October 2023 (fig 2). Meanwhile, the prevalence of long term non-daily vaping remained relatively stable (between 0.6% and 0.7%) up to May 2021, then increased to 1.6% by October 2023 (fig 2). The difference between the prevalence of long term daily vaping compared with long term non-daily vaping was less pronounced among never smokers (1.5% (1.1% to 2.1%) *v* 0.6% (0.3% to 0.9%) in October 2023; supplementary figure S1). When we investigated changes in vaping frequency among long term vapers in detail (using unmodelled data aggregated by survey year), a notable decrease was observed from 2013/14 to 2022/23 in the proportions who said they were vaping less than weekly (from 18.3% to 5.7%) or at least weekly but less than daily (from 20.6% to 8.9%), and increases in the proportions who said they were vaping ≥12 times a day (from 10.6% to 25.8%) or who did not know (from 6.9% to 18.9%; supplementary table S3).

Trends also differed according to the main type of device used. In July 2016, when device type was first assessed, most long term vapers mainly or exclusively used refillable devices (2.5% of adults) and few mainly or exclusively used disposable devices (0.1% of adults, table 1; estimates by main device type used do not sum to the total prevalence of long term vaping because each device type was modelled separately). By October 2023, similar proportions of vapers mainly or exclusively used refillable or disposable devices (4.6% and 4.9% of adults, respectively; table 1). The prevalence of long term vaping using a disposable device was low (0.1%) between October 2013 and March 2021, then increased rapidly to 4.9% by October 2023 (fig 2). The prevalence of long term vaping using a refillable device increased from 2.5% to 2.7% between October 2013 and September 2017, was stable at 2.7% to September 2019, then increased to 4.6% by October 2023 (fig 2). The prevalence of long term vaping using a pod device was roughly stable (between 0.3% and 0.5%) from October 2013 to March 2021, then increased steadily to 1.0% by October 2023 (fig 2).

Across the study period, 577/6173 long term vapers (9.3%) also used other non-combustible nicotine products (of whom 516 used nicotine replacement therapy, 48 heated tobacco products, and 36 nicotine pouches). Results of sensitivity analyses in which these participants were not counted as long term vapers showed a similar pattern (supplementary table S4 and figures S2 and S3), although absolute estimates of prevalence were slightly lower—for example, the overall estimate for prevalence of long term vaping in October 2023 was 9.1% (95% CI 8.3% to 9.9%) compared with 10.0% (9.2% to 10.9%) in the primary analysis.

### Differences by smoking status, age, gender, and occupational social grade

**Fig 3:**
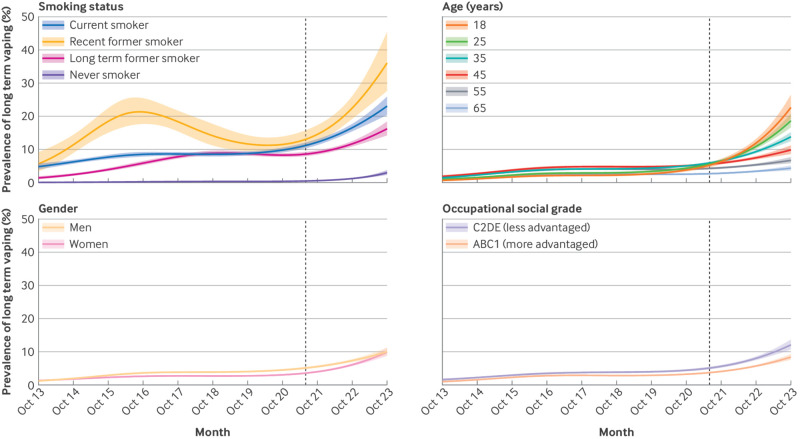
Trends in long term vaping within subgroups of adults in England, October 2013 to October 2023. Lines represent modelled weighted prevalence by monthly survey wave, modelled non-linearly using restricted cubic splines (five knots). Shaded bands represent 95% confidence intervals. Vertical dashed line indicates start of the rise in popularity of vaping using disposable devices in June 2021. Never smokers are those who reported not having been a regular smoker

The increase in long term vaping occurred predominantly among current and former smokers, but a rise also occurred among never smokers in more recent years (from <0.5% to March 2021 to 3.0% by October 2023; table 2, fig 3).

**Table 2:** Modelled estimates of changes in prevalence of long term vaping and long term vaping using a disposable device within subgroups of adults in England

	Any long term vaping, % (95% CI)*			Long term vaping using disposable device, % (95% CI)*	
	October 2013	October 2023		July 2016	October 2023
**Smoking status**					
Never smoker	0.1 (0.0 to 0.2)	3.0 (2.3 to 3.8)		0.0 (0.0 to 0.3)	1.8 (1.2 to 2.5)
Former smoker:					
Long term (≥1 year)	1.4 (1.0 to 1.9)	16.2 (14.2 to 18.4)		0.1 (0.0 to 0.5)	4.4 (3.1 to 6.0)
Recent (<1 year)	5.7 (3.4 to 9.2)	36.1 (27.6 to 45.4)		0.3 (0.0 to 3.9)	24.9 (15.9 to 36.8)
Current smoker	4.8 (4.0 to 5.8)	23.1 (20.4 to 25.9)		0.4 (0.2 to 0.9)	13.6 (11.1 to 16.6)
**Age (years)†**					
18	0.7 (0.4 to 1.0)	22.7 (19.2 to 26.5)		0.0 (0.0 to 0.2)	19.1 (14.8 to 24.4)
25	1.0 (0.7 to 1.3)	18.6 (16.7 to 20.8)		0.0 (0.0 to 0.2)	12.2 (10.2 to 14.6)
35	1.5 (1.2 to 1.8)	13.8 (12.5 to 15.2)		0.1 (0.0 to 0.2)	6.3 (5.1 to 7.7)
45	1.9 (1.5 to 2.3)	9.9 (8.6 to 11.2)		0.2 (0.1 to 0.4)	3.2 (2.4 to 4.3)
55	1.7 (1.4 to 2.1)	6.7 (5.8 to 7.7)		0.2 (0.1 to 0.6)	1.8 (1.3 to 2.4)
65	1.1 (0.9 to 1.4)	4.3 (3.6 to 5.2)		0.2 (0.1 to 0.4)	1.0 (0.7 to 1.6)
**Gender**					
Men	1.2 (1.0 to 1.5)	10.1 (8.9 to 11.3)		0.1 (0.0 to 0.2)	5.1 (4.1 to 6.3)
Women	1.3 (1.1 to 1.6)	9.9 (8.8 to 11.2)		0.2 (0.1 to 0.4)	4.7 (3.8 to 5.8)
**Occupational social grade**					
ABC1 (more advantaged)	1.0 (0.8 to 1.2)	8.4 (7.6 to 9.3)		0.1 (0.0 to 0.3)	4.1 (3.4 to 5.0)
C2DE (less advantaged)	1.6 (1.3 to 2.0)	12.1 (10.6 to 13.7)		0.1 (0.0 to 0.3)	5.8 (4.6 to 7.3)

CI=confidence interval.

* Data for October 2013, July 2016, and October 2023 are weighted estimates of prevalence in these months from logistic regression, with survey month modelled non-linearly using restricted cubic splines (five knots). October 2013 and October 2023 were the first and last months in the time series, and July 2016 was the first month in which the main device type used by vapers was assessed.

† The model used to derive these estimates included data from participants of all ages, not only those who were aged exactly 18, 25, 35, 45, 55, or 65 years.

Changes in long term vaping were similar across ages up to 2019, but prevalence then increased more rapidly among young adults than older adults (fig 3), resulting in a strong inverse age gradient in long term vaping (eg, reaching 22.7% among 18 year olds *v* 4.3% among 65 year olds by October 2023; table 2). This pattern of results persisted after adjustment for psychological distress (supplementary figure S4). A similar inverse age gradient was also observed among never smokers (eg, reaching 16.1% among 18 year olds *v* 0.3% among 65 year olds; supplementary table S5 and figure S5).

The prevalence of long term vaping initially rose slightly more quickly among men, and as a result was significantly higher among men than women between June 2015 and December 2022 (fig 3). However, prevalence then increased more quickly among women than men from late 2021, closing this gap (fig 3). As of October 2023, no significant difference was observed in the prevalence of long term vaping between men and women (10.1% and 9.9%, respectively; table 2).

The prevalence of long term vaping was consistently higher among those from less advantaged social grades compared with more advantaged social grades, but time trends were similar (fig 3).

**Fig 4:**
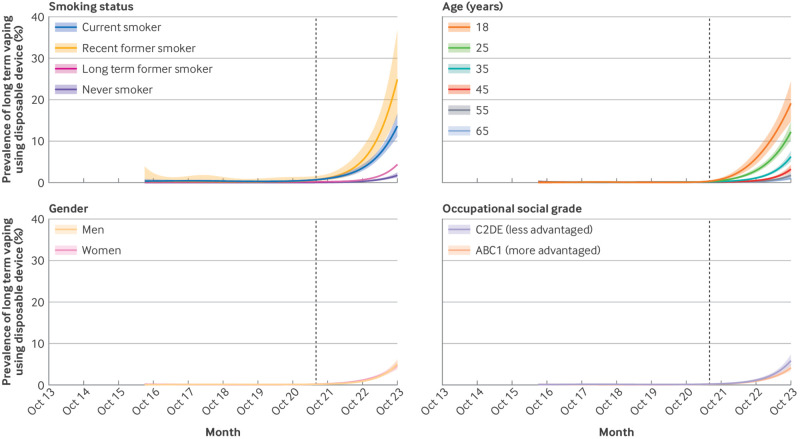
Trends in long term vaping using disposable devices within subgroups of adults in England, July 2016 to October 2023. Lines represent modelled weighted prevalence by monthly survey wave, modelled non-linearly using restricted cubic splines (five knots). Shaded bands represent 95% confidence intervals. Vertical dashed line indicates start of the rise in popularity of vaping using disposable devices in June 2021. Never smokers are those who reported not having been a regular smoker

Recent changes in long term vaping using disposable devices by smoking status, age, gender, and occupational social grade followed similar patterns to those observed for changes in any long term vaping (table 2, fig 4).

## Discussion

In England, the prevalence of long term (>6 months) vaping increased substantially from 2013 to 2023. In October 2013, when e-cigarettes were still relatively new and less effective in delivering nicotine than current devices, around one in 80 adults was a long term vaper. This number increased to one in 30 adults by mid-2017 and was stable for several years. Then a rapid rise started in 2021, and by October 2023 one in 10 adults in England reported having been vaping for more than six months. The rise in long term vaping was largely driven by an increase in long term daily vaping. The absolute increases in long term vaping were most pronounced among people with a history of regular smoking, but an increase also occurred among people who had never regularly smoked. Growth was also most pronounced in young adults, including among those who had never regularly smoked. This pattern persisted after adjustment for rises in psychological distress over this period.^25^ Between October 2013 and March 2021, most long term vapers mainly or exclusively used refillable e-cigarettes, and few used disposable devices. However, the prevalence of long term vaping using disposable devices subsequently rose rapidly, and by October 2023 similar proportions of adults mainly or exclusively used disposable and refillable devices. The prevalence of long term vaping using a pod device increased between March 2021 and October 2023 but remained relatively rare.

### Comparison with other studies

The timing of the sharp rise in long term vaping coincided with the rising popularity of new disposable e-cigarettes from Spring 2021.^7^ ^8^ This suggests that the recent increase in vaping among adults—particularly young adults—in England does not just reflect an increase in experimental use, but rather that a substantial number of those taking up vaping are going on to vape long term. Our data also suggest that most of those vaping long term are vaping daily, including more than two thirds of those who have never regularly smoked (ie, it is not just infrequent use over a long period). In addition, although in recent years small increases in long term vaping using refillable and pod devices have occurred, the increase in long term vaping using disposable devices was substantially larger. This indicates that many vapers are now continuing to use disposable devices over the long term, rather than transitioning to rechargeable devices.

We observed notable differences in trends in long term vaping by age and smoking status. The recent and rapid rise in long term vaping followed a clear inverse age gradient that mirrored the pattern we have seen for the prevalence of current vaping using disposable devices over this period.^7^ ^8^ The rise occurred predominantly among current and former smokers, but an increase in long term vaping also occurred among those who had never regularly smoked. This is not necessarily a cause for concern if vaping is diverting people who would have otherwise smoked towards a less harmful nicotine product. Simulation modelling of trends in smoking and vaping among adolescents in the US suggests a substantial diversion effect is likely.^26^ However, if these people would not have otherwise taken up smoking, then taking up vaping as a regular habit will expose them to greater harm than if they had neither vaped nor smoked.^3^ With a growing proportion of people using e-cigarettes, it becomes increasingly unlikely that all would have used cigarettes instead, given the declining trends in prevalence of cigarette smoking.^27^ Across our study period, national estimates of smoking prevalence among adults in England fell from 18.8% in 2013 to 12.9% in 2022.^27^ Recent data indicate a rise in the proportion of young adults using inhaled nicotine (ie, vaping or smoking) since disposable e-cigarettes became popular in the absence of an acceleration in the decline in smoking, although declines in smoking appear to have been most pronounced in age groups with the largest increase in vaping.^27^ ^28^ Our current study shows that the proportion of young adults vaping long term is now higher than smoking rates have been in this age group since 2017.^27^ A similar pattern has been documented among adolescents, with rates of current vaping among 11-17 year olds in Great Britain in 2023 higher than smoking rates in this age group have been for more than a decade.^5^

Our definition of long term vaping was use of e-cigarettes for a period of more than six months and was an operational definition to indicate possible dependent use. It is not necessarily indicative of a length of time at which we would expect substantial harms from vaping to have already accumulated. In discussions about the risks associated with e-cigarettes, a key concern is that the harms of long term use are not yet fully understood—in this context, long term use refers to decades rather than the much shorter duration of use focused on in our analysis. Even most of the well established harmful effects of tobacco smoking on health outcomes only become apparent after years and decades, rather than months, of use. Existing evidence suggests the long term health risks of vaping are likely to be substantially lower than the risks of smoking.^3^

### Policy implications

Our findings raise two key issues with implications for policy. Firstly, disposable e-cigarettes appear to be attracting young adults to establish long term e-cigarette use. Long term vaping among young adults has increased exceptionally since 2021, and as of October 2023 it did not yet show signs of stopping. This adds weight to calls for tighter regulation of vaping products to reduce their appeal to young people^29^ and highlights the urgency of this action. Disposable e-cigarettes might appeal to young people for a variety of reasons, including affordability, sleek design and branding, attractive in-store displays, and ease of access.^29^ ^30^ ^31^ Any policies intended to reduce young people’s interest in vaping, however, must be carefully considered in case they discourage people from using e-cigarettes as a smoking cessation aid.^32^ ^33^ Simultaneously meeting both goals will likely present challenges. The UK government is considering restricting e-cigarette flavours, restricting branding across all e-cigarette products and packaging, and restricting the visibility and promotion of e-cigarettes in shops.^34^ Although sweet and fruit flavours likely increase the appeal of e-cigarettes to young people,^35^ they are also important to adult smokers,^36^ ^37^ so restricting them may discourage smokers from switching to vaping. Consistent with this, recent data from the US suggest that restrictions on the sale of flavoured e-cigarettes are associated with increased cigarette sales.^38^ Similarly, restricting the visibility and promotion of e-cigarettes in shops, while helping to discourage use by adolescents and young adults, might also lead to some degree of reduction in smokers’ use of e-cigarettes in quit attempts. This could potentially be mitigated by public health campaigns promoting the use of e-cigarettes as a cessation aid, including at the point of sale. Explicitly targeting e-cigarette advertising towards older smokers—who are less likely to be using e-cigarettes and could benefit most from using them to stop smoking—could also be helpful in positioning these products as something that older people use and making them less attractive to adolescents and young adults without a smoking history. An additional measure the government is reported to be considering is to apply a tax to e-cigarettes to increase the price and make them less affordable to young people.^39^ When raising the price of e-cigarettes, it would be important to make sure that they remain less expensive than cigarettes,^40^ ^41^ to mitigate unintended reductions in their use for smoking cessation. US data suggest that higher tax rates on e-cigarette products are associated with reductions in use of e-cigarettes but increases in cigarette smoking among young adults.^42^

The second issue is that as of October 2023, half of long term vapers are mainly or exclusively using disposable devices (and most are using them every day), which has a substantial impact on the environment. A typical disposable e-cigarette is designed for single use and contains plastic, rubber, and copper, and a lithium battery. Although some parts (eg, the battery) can be widely recycled, other parts (eg, rubber) cannot, and devices are not generally designed to be taken apart easily. If they are not disposed of correctly, they can potentially release plastic, electronic, and hazardous chemical waste into the environment.^14^ There have been calls for an outright ban on disposable e-cigarettes,^43^ ^44^ ^45^ and the UK government recently announced its intention to follow this course of action.^34^ However, these products offer advantages for certain groups (eg, those with severe mental illness, who may find them easier to use^46^ ^47^) and in certain situations (eg, for short term use when a person has forgotten their rechargeable e-cigarette or the battery has run out while out). Raising the upfront cost of disposable e-cigarettes to more than that of the cheapest reusable e-cigarettes (eg, through an excise tax), thus making reusable e-cigarettes a more cost-effective alternative, may encourage long term vapers to transition to less environmentally damaging products over time,^29^ while allowing disposable devices to remain available to those who need them more.

### Directions for future research

Our findings also raise questions that could be addressed in future research. Firstly, have similar increases occurred in long term vaping among adolescents, or in other countries where disposable e-cigarettes have become the dominant product used? The 2023 Action on Smoking and Health (ASH) Smokefree GB Youth Survey found that while 20.5% of 11-17 year olds surveyed had tried vaping, most of this was experimentation (11.6% tried once or twice) or occasional use (3.9% use less than once a week).^5^ However, recent data from the International Tobacco Control Youth Tobacco and Vaping Survey suggest that increases in vaping prevalence among young people (aged 16-19 years) in England, the US, and Canada over recent years have been accompanied by greater levels of dependence on vaping (including increases in perceived addiction, urges, and frequency of use),^48^ which could lead to an increase in long term use.

Secondly, to what extent has the covid-19 pandemic contributed to the rising prevalence of vaping among young people? Although our data clearly show a sharp rise in long term vaping among adults since disposable e-cigarettes became popular (including in the segmented regression model, which adjusted for the onset of the pandemic), visual inspection of unmodelled data points also suggests a modest increase around the start of the pandemic (fig 1). Although some of this is likely to be attributable to an increase in smoking cessation during the acute phase of the pandemic,^49^ ^50^ other factors may have contributed to the rise—particularly among young people (eg, stresses associated with reduced social contact, and disruption to school and university study and exam schedules).

Finally, what are the motives for long term vaping among current smokers? Our data suggest that more than one in three recent former smokers and one in six long term former smokers is a long term vaper. It is likely that a sizable proportion of each group quit smoking by vaping, since e-cigarettes are the most popular cessation aid in England (used in around a third of quit attempts),^4^ are effective for helping people quit,^1^ ^2^ and have been associated with reductions in smoking prevalence at population level.^51^ ^52^ ^53^ It would be interesting to know more about why the 23% of current smokers who are long term vapers use e-cigarettes—for example, are they trying to quit smoking or reduce their cigarette consumption for harm reduction, or are they using e-cigarettes situationally,^54^ possibly sustaining their smoking—and to what extent do these motives differ by socioeconomic position or other sociodemographic or smoking related factors?

### Strengths and limitations of this study

Strengths of this study include the large, representative sample and repeated monthly assessments over a 10 year period. Limitations relate to the study design and measures. Although we have speculated that the rise in popularity of disposable e-cigarettes may have contributed to the substantial increase in long term vaping we observed, the correlational design means we cannot definitively infer causality. We used a hybrid sampling approach rather than random probability sampling, although comparisons with other sources—national probability surveys and sales figures—suggest that the survey recruits a nationally representative sample and produces similar estimates of key smoking related variables.^17^ ^18^ Our sample focused on adults, so the data do not offer any insights into the extent to which the recent rise in vaping among adolescents reflects long term use versus experimental use.

The assessment of vaping in the Smoking Toolkit Study is somewhat unusual (described in the methods section) because it was not initially a priority when first added in 2011, and subsequently the team have not wanted to lose long term comparability of the trends. However, we see close alignment in estimates of vaping prevalence with other nationally representative surveys in England that use more straightforward language. The items assessing vaping identified participants who reported using an e-cigarette for any reason. Although we did not restrict the definition of long term vaping to vaping using nicotine, because nicotine content was not assessed in every wave and not everyone knows the nicotine content of the device they use, most (87% of those asked) said their usual device contains nicotine.

The main device type used by vapers was not assessed before July 2016, so we were unable to model trends in long term vaping by device type across the entire period. In addition, no definition was provided for “mainly used” when asked about device type, so it was open to participants’ interpretation. The items assessing vaping frequency and main device type only captured current behaviour, so our data do not offer insight into how these variables had changed within individuals with increasing duration of vaping. While this may have biased absolute estimates of prevalence, it should not affect time trends observed across individuals.

The item assessing vaping duration asked how long people who reported using e-cigarettes (or other nicotine products) had been using “this nicotine replacement product or these products,” which may have led to underreporting among participants who did not consider e-cigarettes to be a form of nicotine replacement (eg, never smokers). The Smoking Toolkit Study has, however, produced similar estimates of current vaping prevalence to other national surveys.

The measures of duration and frequency of use were not specific to vaping but covered all non-combustible nicotine products participants reported using, but most long term vapers in the sample reported only using e-cigarettes, and the results of a sensitivity analysis restricting the definition of long term vaping to those who did not also use other products showed a similar pattern.

Finally, our approach to determining smoking status meant that the never smoker group could include some people who smoked for less than a year (although it is likely that many people who previously smoked would select a response option that classified them as a former smoker, given that the question asked which descriptor applied to them best). However, before 2020, this measure indicated a low prevalence of long term vaping among never smokers and much higher prevalence among current and former smokers. Given the considerable change in trends we observed, it seems unlikely that this limitation could explain the pronounced increase in long term vaping. Moreover, even if everyone who reported long term vaping had a history of smoking (whether it was disclosed in response to the item assessing smoking status), this would represent a serious departure from recent trends in smoking that warrants attention.

### Conclusions

The prevalence of long term (>6 months) vaping increased substantially among adults in England from 2013 to 2023. Much of this increase occurred from 2021, coinciding with the rapid rise in popularity of disposable e-cigarettes. Half of long term vapers now mainly or exclusively use disposable devices. The growth has been concentrated among people with a history of regular smoking, but an increase has also occurred among those who have never regularly smoked, especially young adults.

**What is already known on this topic**

Vaping prevalence has increased substantially in England since new disposable electronic cigarettes became popular in mid-2021, particularly among adolescents and young adults
It is not clear how far this reflects an increase in experimental use versus long term, regular use
In addition, little is known about how the types of products used by long term vapers are changing over time

**What this study adds**

Long term vaping has noticeably increased among young adults since 2021, including among those who have never regularly smoked
Half of long term vapers now mainly or exclusively use disposable e-cigarettes, and most are using them every day

## Data Availability

The data and code used for these analyses are available on Open Science Framework (https://osf.io/n2785/), with age provided in bands to preserve participant anonymity.

## Web extra

Extra material supplied by authors

Supplementary information: Items assessing vaping status, additional tables S1-S5 and figures S1-S5

## Notes

Transparency: The authors (the manuscript’s guarantors) affirm that the manuscript is an honest, accurate, and transparent account of the study being reported; that no important aspects of the study have been omitted; and that any discrepancies from the study as originally planned and registered have been explained.

Dissemination to participants and related patient and public communities: We will discuss these findings with our PPI group and ask for input on our press release, briefings, and dissemination strategy. Following this discussion, we plan to disseminate through a press release and social media. We will also share the results directly with key stakeholders (eg, Department of Health and Social Care, Office for Health Improvement and Disparities, and Action on Smoking and Health (ASH)). We have previously co-authored briefings for schools with ASH (https://ash.org.uk/resources/view/addressing-common-myths-about-vaping-putting-the-evidence-in-context) and commented on “ASH guidance on developing school policies on vaping” and “ASH Policy brief on vaping - February 2023,” including on vaping in adolescents. We will work with ASH to consider whether these documents should be updated in light of these new findings, and whether we could contribute to an ASH hosted webinar on the issue.

Provenance and peer review: Not commissioned; externally peer reviewed.
