# Supplementary material for: Trends in long term vaping among adults in England, 2013-23: population based study
Source: BMJ. 2024 Jul 17;386:e079016. doi: 10.1136/bmj-2023-079016 (PMC11253215; doi:10.1136/bmj-2023-079016)
Supplement: Supplementary file 1 — Supplementary information: Items assessing vaping status, additional tables S1-S5 and figures S1-S5 [file jacs079016.ww.pdf]

Items assessing vaping status

The following questions assessed vaping status. For each question, interviewers were prompted to probe fully, asking 'Which others?' until the participant responded that they did not use any other of these products.

Current smokers were asked:

1. Which, if any, of the following are you currently using to help you cut down the amount you smoke?
    a) Nicotine gum
    b) Nicotine replacement lozenges\tablets
    c) Nicotine replacement inhaler
    d) Nicotine replacement nasal spray
    e) Nicotine patch
    f) Electronic cigarette
    g) Nicotine mouthspray
    h) Heat-not-burn cigarette (e.g. iQOS, heatsticks)
    i) Juul
    j) Tobacco-free nicotine pouch/pod or 'white pouches' that you place on your gum (e.g., Zyn, On!, Nordic Spirit, Velo, Lyft, Skruf)
    k) Other (specify)

2. Do you regularly use any of the following in situations when you are not allowed to smoke?
    a) Nicotine gum
    b) Nicotine lozenge
    c) Nicotine patch
    d) Nicotine inhaler\inhalator
    e) Another nicotine product
    f) Electronic cigarette
    g) Nicotine mouthspray
    h) Heat-not-burn cigarette (e.g. iQOS, heatsticks)
    i) Juul
    j) Tobacco-free nicotine pouch/pod or 'white pouches' that you place on your gum (e.g., Zyn, On!, Nordic Spirit, Velo, Lyft, Skruf)
    k) Other (specify)

Past-year smokers (i.e., current smokers and those who quit in the past year) were asked:

1. Can I check, are you using any of the following either to help you stop smoking, to help you cut down or for any other reason at all?
    a) Nicotine gum
    b) Nicotine lozenge
    c) Nicotine patch
    d) Nicotine inhaler\inhalator
    e) Another nicotine product

f) Electronic cigarette
g) Nicotine mouthspray
h) Heat-not-burn cigarette (e.g. iQOS, heatsticks)
i) Juul
j) Other (specify)

Non-smokers were asked:

1. Can I check, are you using any of the following?
    a) Nicotine gum
    b) Nicotine lozenge
    c) Nicotine patch
    d) Nicotine inhaler\inhalator
    e) Another nicotine product
    f) Electronic cigarette
    g) Nicotine mouthspray
    h) Heat-not-burn cigarette (e.g. iQOS, heatsticks)
    i) Juul
    j) Tobacco-free nicotine pouch/pod or 'white pouches' that you place on your gum (e.g., Zyn, On!, Nordic Spirit, Velo, Lyft, Skruf)
    k) Other (specify)

To assess nicotine vaping, those who report using an electronic cigarette in response to any of these questions are asked:

1. Does the electronic cigarette or vaping device you mainly use contain nicotine?
    a) Yes
    b) No
    c) Don’t know

The full questionnaire is available at https://smokinginengland.info/resources/sts-documents.

Table S1. Weighted sample characteristics

	**Whole analysed sample**[1] **(n^2=179,725)**	**Device type subsample**[3] **(n^2=125,751)**
Smoking status		
Never smoker	62.0%	62.0%
Long-term (≥1y) ex-smoker	18.8%	19.3%
Recent (<1y) ex-smoker	1.6%	1.7%
Current smoker	17.6%	17.0%
Missing, *n*[1]	458	426
Age (years)		
Mean (SD)	47.9 (18.6)	48.1 (18.7)
16-24	12.4%	12.2%
25-34	17.2%	17.2%
35-44	16.4%	16.1%
45-54	17.3%	17.2%
55-64	14.6%	14.7%
≥65	22.1%	22.6%
Gender		
Men	48.9%	48.9%
Women	50.9%	50.8%
Other	0.2%	0.3%
Missing, *n*[1]	66	66
Occupational social grade		
ABC1 (more advantaged)	54.4%	55.7%
C2DE (less advantaged)	44.6%	44.3%

[1] Adults surveyed October 2013 – October 2023.
[2] Unweighted sample size.
[3] Adults surveyed July 2016 – October 2023 and provided data for analyses by main device type used.
Note: Data are shown as weighted column percentages, unless otherwise specified. There were some missing data (unweighted *n*s indicated in the table); valid percentages are shown for ease of interpretation.

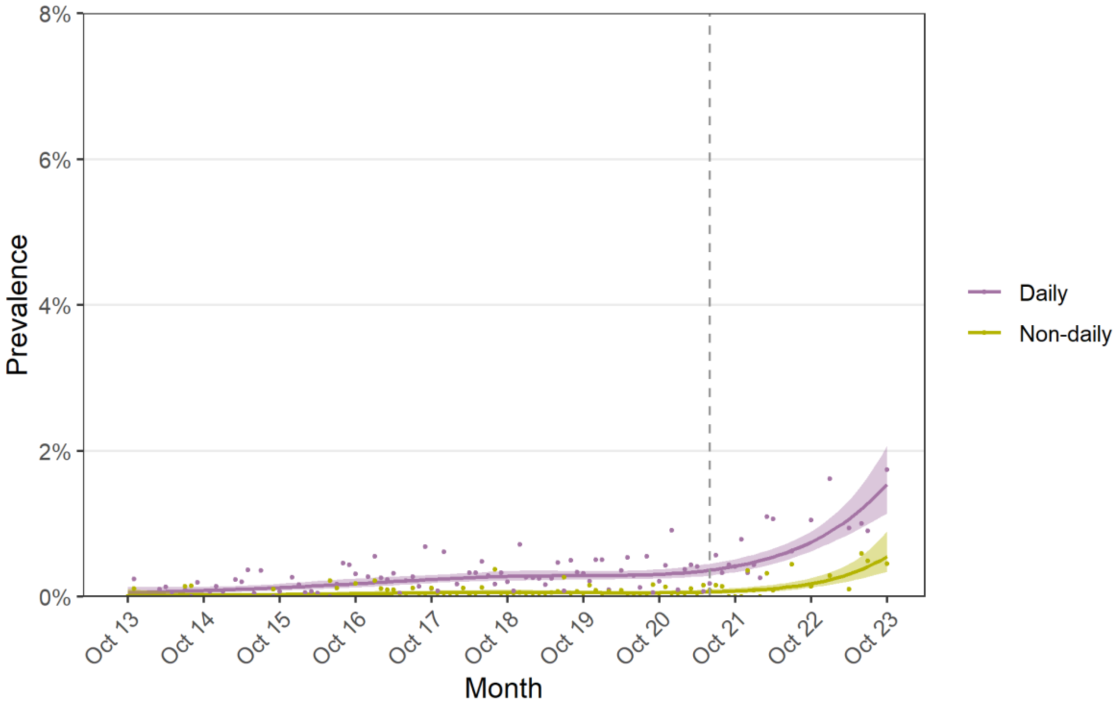

Figure S1. Trends in the prevalence of long-term daily vaping and long-term non-daily vaping among never smokers, October 2013 to October 2023. Lines represent modelled weighted prevalence by monthly survey wave, modelled non-linearly using restricted cubic splines (five knots). Shaded bands represent 95% confidence intervals. Points represent unmodelled weighted prevalence by month. The vertical dashed line indicates the timing of the start of the rise in popularity of disposable vaping in June 2021.

Table S2. Prevalence of long-term vaping by year among adults in England, 2013/14 to 2022/23

	**Prevalence, % [95% CI][1]**									
	**2013/14**	**2014/15**	**2015/16**	**2016/17**	**2017/18**	**2018/19**	**2019/20**	**2020/21**	**2021/22**	**2022/23**
Overall										
Long-term vaping	1.6	2.5	2.9	3.3	3.5	3.2	3.9	4.9	4.9	8.6
	[1.4 to 1.8]	[2.2 to 2.7]	[2.6 to 3.2]	[3.0 to 3.5]	[3.2 to 3.8]	[2.9 to 3.5]	[3.6 to 4.2]	[4.5 to 5.2]	[4.5 to 5.3]	[7.9 to 9.3]
By vaping frequency										
Long-term daily vaping	0.9	1.5	2.0	2.4	2.5	2.4	2.8	3.3	3.4	5.7
	[0.7 to 1]	[1.3 to 1.7]	[1.8 to 2.3]	[2.2 to 2.6]	[2.3 to 2.8]	[2.1 to 2.6]	[2.5 to 3.0]	[3.0 to 3.6]	[3.0 to 3.7]	[5.2 to 6.3]
Long-term non-daily vaping	0.6	0.8	0.7	0.6	0.7	0.6	0.7	0.9	0.8	1.3
	[0.5 to 0.7]	[0.7 to 1.0]	[0.6 to 0.8]	[0.5 to 0.7]	[0.6 to 0.9]	[0.4 to 0.7]	[0.5 to 0.8]	[0.7 to 1.0]	[0.6 to 1.0]	[1.0 to 1.5]
By main device type used[2]										
Long-term disposable vaping	-	-	-	0.1	0.1	0.1	0.1	0.1	0.7	2.9
				[0.1 to 0.2]	[0.1 to 0.2]	[0.1 to 0.2]	[0.0 to 0.1]	[0.1 to 0.2]	[0.5 to 0.8]	[2.5 to 3.3]
Long-term refillable vaping	-	-	-	2.7	2.8	2.6	3.1	3.9	3.3	4.6
				[2.5 to 3.0]	[2.5 to 3.0]	[2.3 to 2.8]	[2.8 to 3.4]	[3.6 to 4.2]	[2.9 to 3.7]	[4.1 to 5.1]
Long-term pod vaping	-	-	-	0.4	0.5	0.5	0.5	0.6	0.7	1.0
				[0.3 to 0.5]	[0.4 to 0.7]	[0.4 to 0.6]	[0.4 to 0.6]	[0.5 to 0.7]	[0.6 to 0.9]	[0.7 to 1.2]

[1] Data are weighted proportions, aggregated by survey year (October through September).

[2] Estimates of prevalence by vaping frequency and main device type used do not sum to the overall prevalence because they do not include participants who responded 'don't know' or who did not provide a response.

[3] The main device type used was not assessed before July 2016.

Table S3. Frequency of vaping among long-term vapers, 2013/14 to 2022/23

	Prevalence, % [95% CI][1]									
	**2013/14**	**2014/15**	**2015/16**	**2016/17**	**2017/18**	**2018/19**	**2019/20**	**2020/21**	**2021/22**	**2022/23**
Once a day	6.5 [4.1 to 10.1]	7.2 [5.0 to 10.5]	6.4 [4.5 to 9.0]	4.2 [2.8 to 6.2]	5.4 [3.9 to 7.6]	4.7 [3.3 to 6.7]	4.1 [2.7 to 6.1]	5.9 [4.4 to 7.8]	6.4 [4.6 to 8.8]	3.1 [2.1 to 4.6]
Twice a day	5.9 [3.6 to 9.4]	4.2 [2.6 to 6.7]	3.2 [2.0 to 5.2]	6.2 [4.5 to 8.5]	4.1 [2.8 to 6.0]	4.9 [3.4 to 7.2]	3.9 [2.7 to 5.7]	5.8 [4.3 to 7.8]	5.9 [4.0 to 8.6]	4.2 [3.0 to 6.0]
3-4 times a day	9.4 [6.5 to 13.2]	8.7 [6.3 to 11.9]	8.4 [6.1 to 11.5]	7.3 [5.4 to 9.8]	8.5 [6.5 to 11.1]	8.1 [6.1 to 10.7]	7.2 [5.3 to 9.6]	6.7 [5.1 to 8.7]	7.6 [5.6 to 10.2]	7.8 [5.8 to 10.4]
5-7 times a day	12.4 [9.0 to 16.9]	11.9 [9.0 to 15.5]	13.3 [10.4 to 16.9]	13.9 [11.2 to 17.0]	11.5 [9.1 to 14.4]	12.1 [9.6 to 15]	11.2 [8.9 to 14.1]	9.9 [8.0 to 12.3]	11.6 [9.1 to 14.7]	13.4 [10.8 to 16.5]
8-11 times a day	9.3 [6.3 to 13.6]	10.6 [7.6 to 14.4]	14.0 [11.2 to 17.4]	12.4 [9.9 to 15.5]	14.6 [11.9 to 17.8]	14.0 [11.2 to 17.3]	14.8 [12.1 to 17.9]	14.4 [12.0 to 17.1]	12.5 [9.9 to 15.6]	12.2 [9.8 to 15.2]
12+ times a day	10.6 [7.5 to 14.9]	18.7 [15.0 to 23.0]	24.7 [20.8 to 29.0]	29.4 [25.7 to 33.4]	28.9 [25.3 to 32.7]	30.4 [26.6 to 34.5]	29.7 [26.1 to 33.6]	25.7 [22.7 to 29.0]	24.4 [20.7 to 28.5]	25.8 [22.3 to 29.6]
Not every day but at least once a week	20.6 [16.1 to 26.1]	20.3 [16.7 to 24.6]	11.5 [8.9 to 14.7]	10.4 [8.2 to 13.2]	13.6 [11.1 to 16.5]	9.0 [6.8 to 11.7]	10.7 [8.4 to 13.5]	10.8 [8.8 to 13.2]	11.6 [9.0 to 14.9]	8.9 [6.9 to 11.4]
Not every day and less than once a week	18.3 [14.2 to 23.3]	13.0 [10.1 to 16.6]	12.0 [9.3 to 15.4]	8.9 [6.8 to 11.8]	7.6 [5.7 to 10.0]	8.3 [6.2 to 11.1]	6.3 [4.7 to 8.5]	7.4 [5.8 to 9.4]	5.1 [3.5 to 7.4]	5.7 [4.1 to 7.9]
Don’t know	6.9 [4.5 to 10.6]	5.3 [3.5 to 8.1]	6.4 [4.6 to 8.9]	7.3 [5.3 to 9.8]	5.8 [4.2 to 8.0]	8.5 [6.1 to 11.6]	12.0 [9.5 to 15.0]	13.4 [11.2 to 15.9]	14.9 [12.1 to 18.1]	18.9 [15.7 to 22.5]

[1] Data are weighted proportions, aggregated by survey year (October through September).

Table S4. Modelled estimates of the prevalence of long-term vaping among adults in England at the start and end of the study period, overall and by main device type and vaping frequency. Sensitivity analysis restricting the definition of long-term vaping to those not using other non-combustible nicotine products.

	Prevalence, % [95% CI][1]		
	**October 2013**	**July 2016**	**October 2023**
Overall			
Long-term vaping	1.0 [0.9 to 1.2]	2.7 [2.6 to 2.9]	9.1 [8.3 to 9.9]
By vaping frequency[2]			
Long-term daily vaping	0.5 [0.4 to 0.7]	1.9 [1.8 to 2.1]	6.0 [5.4 to 6.7]
Long-term non-daily vaping	0.4 [0.3 to 0.6]	0.6 [0.6 to 0.7]	1.4 [1.1 to 1.7]
By main device type used[1]			
Long-term disposable vaping	-	0.1 [0.0 to 0.2]	4.4 [3.7 to 5.1]
Long-term refillable vaping	-	2.3 [2.0 to 2.6]	4.3 [3.7 to 5.0]
Long-term pod vaping	-	0.3 [0.2 to 0.4]	1.0 [0.7 to 1.3]

CI, confidence interval.

[1] Data for October 2013, July 2016, and October 2023 are weighted estimates of prevalence in these months from logistic regression with survey month modelled non-linearly using restricted cubic splines (five knots). October 2013 and October 2023 were the first and last months in the time series and July 2016 was the first month in which the main device type used by vapers was assessed.

[2] Prevalence estimates by vaping frequency and main device type used do not sum to the total prevalence of long-term vaping because there were some missing data (either because participants did not respond or responded 'don't know') and each trend was modelled separately.

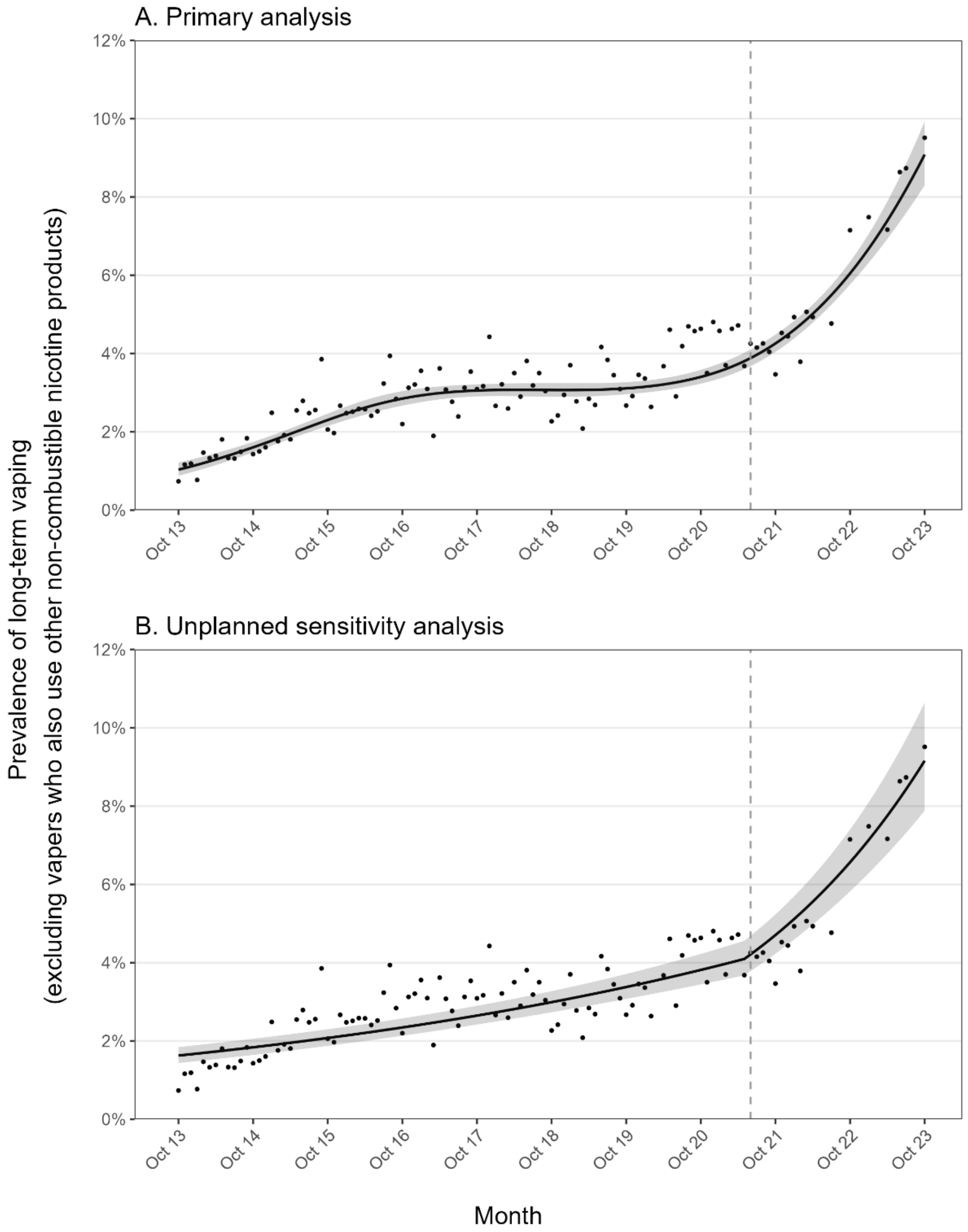

Figure S2. Trends in long-term vaping among adults in England, October 2013 to October 2023. Sensitivity analysis restricting the definition of long-term vaping to those not using other non-combustible nicotine products.

Panel A shows results of the primary model, which modelled survey wave non-linearly using restricted cubic splines (five knots). Panel B shows the results of an unplanned sensitivity analysis, which used a segmented regression approach to model associations of the start of the rise in popularity of disposable e-cigarettes with a change in the trend of long-term vaping (adjusted for seasonality and the onset of the Covid-19 pandemic). Lines represent modelled weighted prevalence by monthly survey wave. Shaded bands represent 95% confidence intervals. Points represent unmodelled weighted prevalence by month. The vertical dashed line indicates the timing of the start of the rise in popularity of disposable vaping in June 2021.

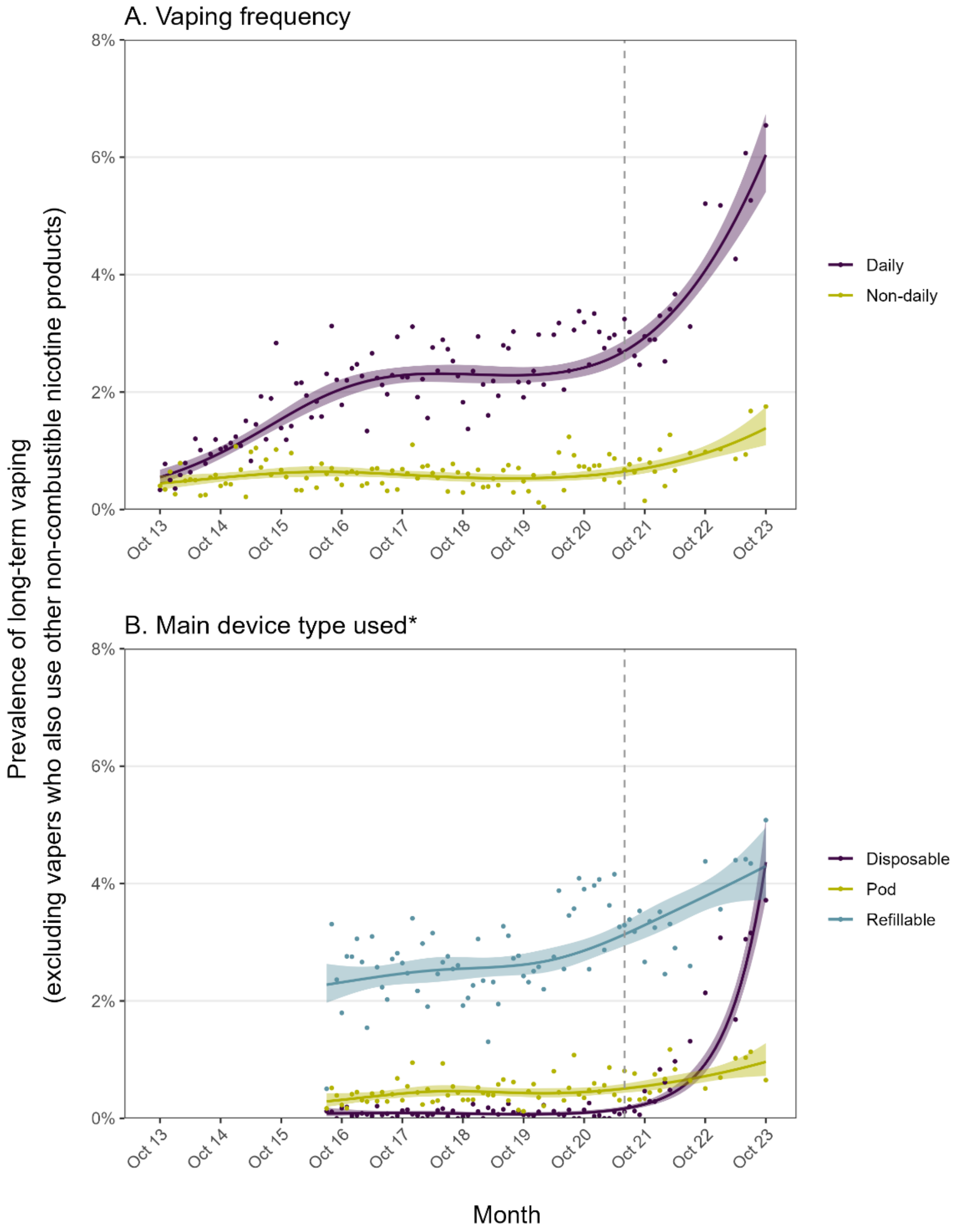

Figure S3. Trends in long-term vaping by frequency and main device type among adults in England, October 2013 to October 2023. Sensitivity analysis restricting the definition of long-term vaping to those not using other non-combustible nicotine products.

Panels show trends in the prevalence of long-term (>6 months) vaping among adults in England by (A) vaping frequency (daily/non-daily) and (B) the main device type used (disposable/refillable/pod). Lines represent modelled weighted prevalence by monthly survey wave, modelled non-linearly using restricted cubic splines (five knots). Shaded bands represent 95% confidence intervals. Points represent unmodelled weighted prevalence by month. The vertical dashed line indicates the timing of the start of the rise in popularity of disposable vaping in June 2021. *The main device type used was not assessed before July 2016. Note that prevalence estimates by vaping frequency and main device type used do not sum to the total prevalence of long-term vaping because there were some missing data (either because participants did not respond or responded 'don't know') and each trend was modelled separately.

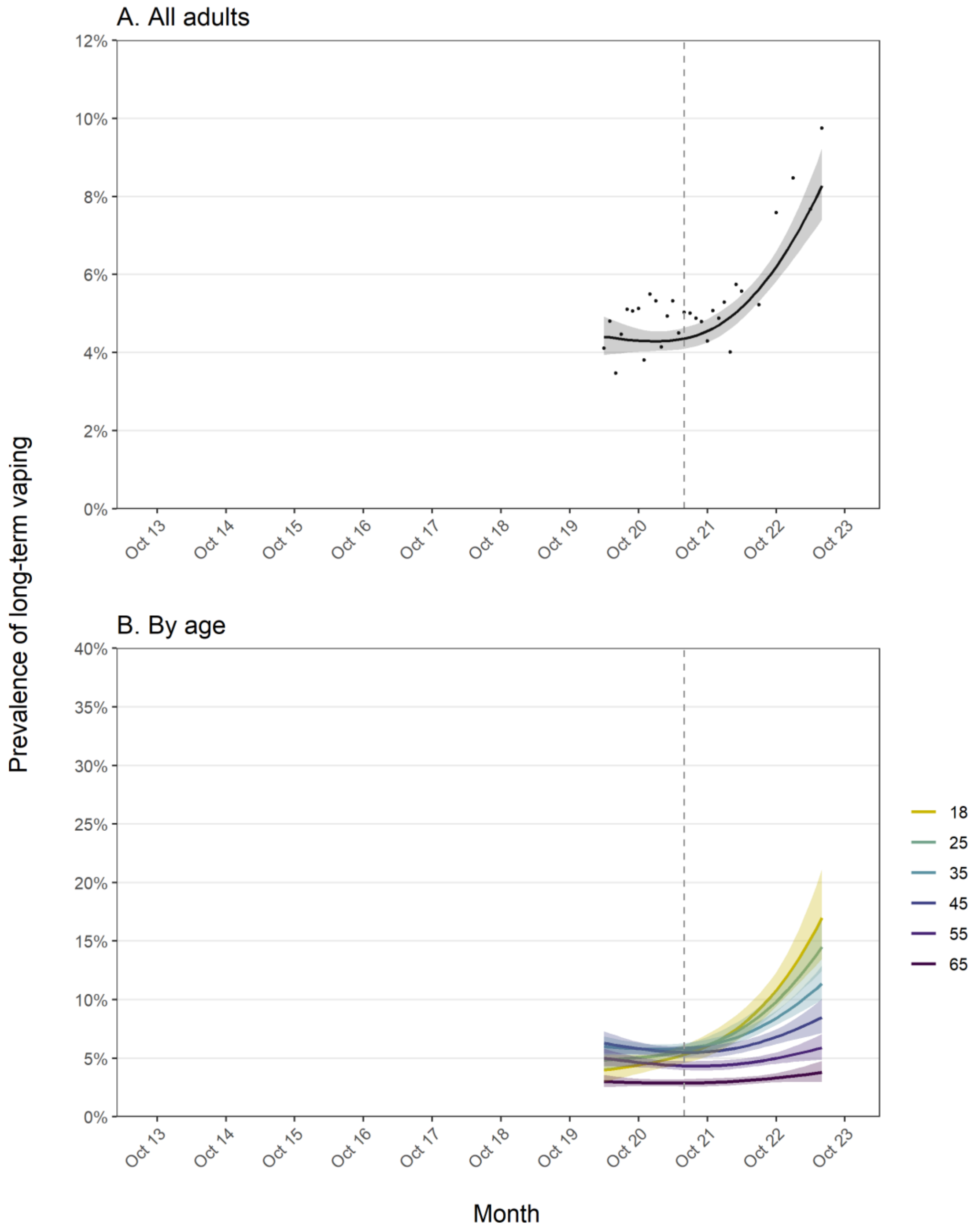

Figure S4. Trends in long-term vaping, overall and by age, April 2020 to June 2023 – adjusting for psychological distress

Panels show trends in the prevalence of long-term (>6 months) vaping among adults in England, (A) overall and (B) by age, with adjustment for psychological distress (K6 score). Lines represent modelled weighted prevalence by monthly survey wave, modelled non-linearly using restricted cubic splines (three knots). Shaded bands represent 95% confidence intervals. Points represent unmodelled weighted prevalence by month. The vertical dashed line indicates the timing of the start of the rise in popularity of disposable vaping in June 2021. Note that the y axis differs between the two panels so that they match the scales used in Figure 1A (all adults) and Figure 3B (by age) to facilitate comparison across trends with and without adjustment for psychological distress.

Table S5. Modelled estimates of changes in the prevalence of long-term vaping among never smokers by age

	Long-term vaping, % [95% CI][1]	
	**October 2013**	**October 2023**
Age (years)[2]		
18	0.0 [0.0 to 0.0]	16.1 [11.1 to 22.7]
25	0.0 [0.0 to 0.1]	8.1 [6.3 to 10.4]
35	0.1 [0.0 to 0.2]	2.9 [2.0 to 4.4]
45	0.2 [0.1 to 0.6]	1.1 [0.6 to 2.1]
55	0.2 [0.1 to 0.4]	0.5 [0.3 to 1.0]
65	0.0 [0.0 to 0.2]	0.3 [0.1 to 0.6]

CI, confidence interval.

[1] Data for October 2013 and October 2023 are weighted estimates of prevalence in these months from logistic regression with survey month modelled non-linearly using restricted cubic splines (five knots). October 2013 and October 2023 were the first and last months in the time series.

[2] Note that the model used to derive these estimates included data from participants of all ages, not only those who were aged exactly 18, 25, 35, 45, 55, or 65 years.

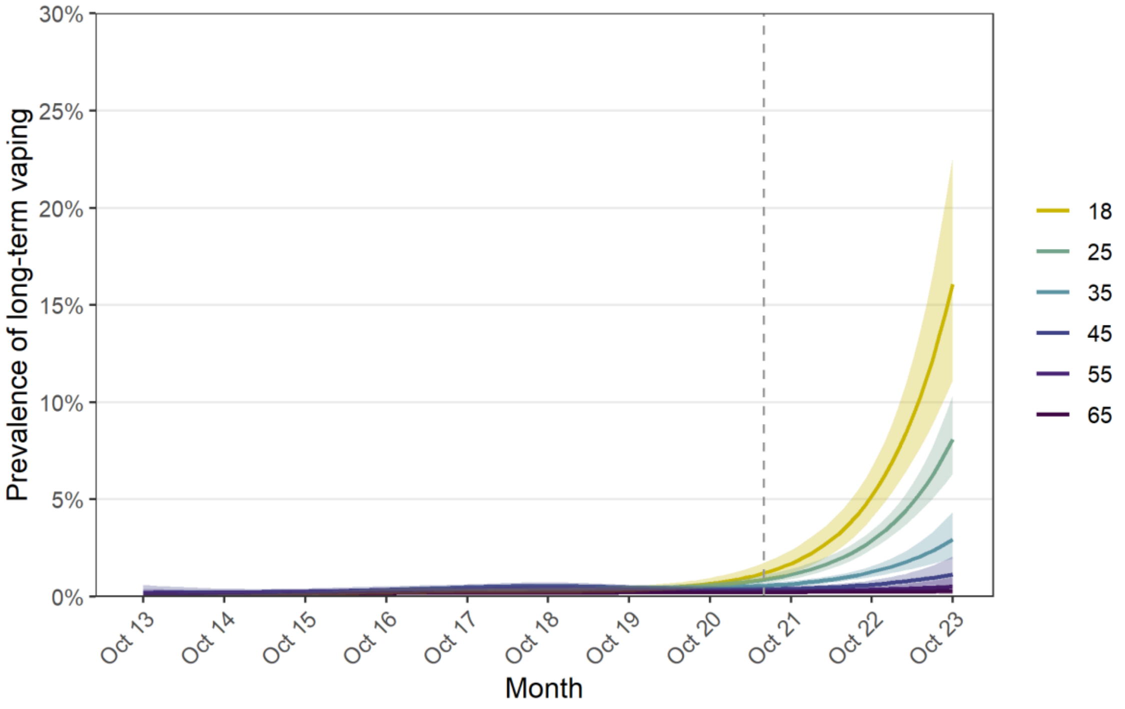

Figure S5. Trends in long-term vaping among never smokers by age, October 2013 to October 2023

The figure shows trends in the prevalence of long-term (>6 months) vaping among adults in England who have never regularly smoked, by age. Lines represent modelled weighted prevalence by monthly survey wave, modelled non-linearly using restricted cubic splines (five knots). Shaded bands represent 95% confidence intervals. The vertical dashed line indicates the timing of the start of the rise in popularity of disposable vaping in June 2021.
